# Illness Uncertainty in Pediatric Irritable Bowel Syndrome: A Pilot Study

**DOI:** 10.3390/children13081106

**Published:** 2026-08-19

**Authors:** Kaitlyn L. Gamwell, Abigail S. Robbertz, James L. Peugh, Neha Santucci, Kahleb Graham, Kevin A. Hommel

**Affiliations:** 1 Department of Pediatrics, School of Medicine, University of Michigan, Ann Arbor, MI 48109, USA; 2 Division of Behavioral Medicine and Clinical Psychology, Department of Pediatrics, Cincinnati Children’s Hospital Medical Center, Cincinnati, OH 45229, USA; 3 Department of Pediatrics, College of Medicine, University of Cincinnati, Cincinnati, OH 45221, USA; 4 Division of Gastroenterology, Hepatology and Nutrition, Department of Pediatrics, Cincinnati Children’s Hospital Medical Center, Cincinnati, OH 45229, USA

**Keywords:** illness uncertainty, pediatric irritable bowel syndrome (IBS), cognitive appraisals, pilot study, clinical signals

## Abstract

**Highlights:**

**What are the main findings?**

- Exploratory indirect effects demonstrated clinical signals for the relationship between illness intrusiveness, uncertainty, and mood, pain related coping, and functional disability.

**What are the implications of the main findings?**

- Illness uncertainty is a key variable in pediatric IBS and future adequately powered studies should use the findings from this study as a platform to build from.
- Targeting illness uncertainty in future interventions may lead to improved psy-chosocial and health related outcomes for youth with IBS.

**Abstract:**

Background/Objectives: Illness uncertainty is inherent in irritable bowel syndrome (IBS); however, the factors that contribute to it and outcomes impacted by it have yet to be assessed in pediatric IBS. As such, the current longitudinal pilot study assessed the relationship between disease characteristics, illness appraisals, mood, and health-related outcomes. Methods: 50 youth (ages 10–18) diagnosed with IBS completed self-report measures across three timepoints. Path analysis models specifying illness uncertainty as the mediator were constructed and assessed with Mplus using Bayesian Estimation. Results: Time-2 illness uncertainty significantly predicted time-3 depressive and anxiety symptoms, higher functional disability, and lower levels of problem- and emotion-focused coping. Exploratory indirect effects demonstrating clinical signals were also identified. Conclusions: Future observational studies with larger samples are needed to further investigate illness appraisals’ relationships with adjustment and health-related outcomes in pediatric IBS. This is a fruitful avenue to identify modifiable areas of targeted intervention.

## 1. Introduction

Pediatric irritable bowel syndrome (IBS), a Disorder of Gut–Brain Interaction (DGBI), is becoming increasingly prevalent in youth. IBS is a chronic health condition that is characterized by intermittent gastrointestinal (GI) symptoms such as stomach pain, constipation, diarrhea, nausea, gas, and increased visceral sensitivity [1]. Due to the intensity of and increased sensitivity to symptoms, particularly pain, individuals with IBS often experience associated functional disability as an outcome. This can be seen in impairments with activities of daily living, social/leisure, school/work, etc. (e.g., [1,2,3]). The often difficult and uncertain nature of reaching a diagnosis of IBS and unpredictable recurrent symptom flares make illness uncertainty a hallmark feature of this chronic health condition (e.g., [4]). Part of the inherent uncertainty with the disease course lies in the gut–brain axis, where neurotransmitters and communication between the brain and stomach occur. This axis is thus largely impacted by aspects such as mood and stress, illness beliefs/appraisals, and to some extent, nutrition [1,5,6]. Thus, it is not uncommon for youth who are diagnosed with IBS to have comorbid diagnoses of anxiety and/or depressive disorders, typically presenting prior to the onset of IBS and subsequently worsening with the physical disease manifestation (e.g., [5,6,7,8]).

Given the “bathroom-related” gastrointestinal symptoms of IBS (e.g., flatulence, fecal urgency or incontinence, intestinal noises), the invisible nature of the illness, and the common age of diagnosis being in adolescence, youth may feel embarrassed and/or not comfortable sharing their experience with peers, teachers, or even caregivers due to fear of how it will be perceived (i.e., legitimacy [4]). This can also lead to increased feelings of thwarted belongingness, strained relationships (e.g., [9]), and illness intrusiveness (i.e., IBS-related disruptions to daily activities/interactions (e.g., [10,11])). The previous literature in pediatric inflammatory bowel disease (IBD) and adult IBS has found the profound impact cognitive appraisals (e.g., illness stigma, intrusiveness, uncertainty, thwarted belongingness) and mood (e.g., anxiety and depressive symptoms) have in various combinations with one another and health-related outcomes (e.g., [7,12,13,14]) in these conditions. However, no known studies have assessed the relationship between the aforementioned illness appraisals, mood, and health-related outcomes in pediatric IBS. Guided by Wright, Afari, and Azutra’s 2009 adaptation of Mishel’s Antecedents and Outcomes of the Uncertainty in Illness model [15,16], the current exploratory pilot study aims to address this gap in the literature and provide context for larger studies to build from.

## 2. Materials and Methods

**Participants and Procedures**

Participants were youth aged 10 to 18 years old with a confirmed diagnosis of IBS (via Rome IV criteria) from a gastroenterologist between July and November of 2020. Recruitment of participants occurred at a center for motility in a large midwestern children’s teaching hospital. Potential participants who also had a diagnosis of inflammatory bowel disease, severe cognitive deficits, and/or whose caregiver was not fluent in English at a 6th-grade level were excluded from the current longitudinal pilot study. Youth completed self-report measures about IBS symptoms, coping, illness appraisals, and mood at baseline (T1), 3-month (T2), and 6-month (T3) follow-up visits and were compensated at each timepoint for participation. This study was approved by the Institutional Review Board and adhered to AMA and APA ethical guidelines. All participants consented in a standardized manner, which included written caregiver permission/consent and written or verbal (for youth ages 7–12 years old) child assent.

**Measures**

***Demographics***

At baseline, youth and their caregivers completed a general demographic form to obtain relevant background information about the participant (e.g., age, race, living situation).

***Disease Parameters***

Chart review of the patient’s electronic health records was performed to verify disease characteristics such as IBS type, date of diagnosis, and symptom activity. At each of the three timepoints, youth also completed a brief 6-item questionnaire asking about their pain, disease activity, stool habits, and treatment. This included a visual analog scale (VAS) to assess their average pain rating over the past week, where emotion faces, numbers, and descriptors ranged from 0, no pain (smiley face) to 10, worst pain imaginable (teary sad face).

*The Functional Disability Index—Child* (FDI-C [3]) is a 15-item questionnaire that assesses physical difficulty due to pain across daily life domains (e.g., walking to the bathroom, doing chores, going shopping) over the past few days. Youth indicate how difficult each activity would have been from 0 = No Trouble to 4 = Impossible. Higher totals indicate greater functional disability. The FDI-C has demonstrated good internal consistency in previous pain populations (e.g., [17,18]). In the current study, the FDI-C demonstrated excellent internal consistency (α = 0.93).

***Illness Appraisals***

The 15-item *Visceral Sensitivity Index* [19] was used to assess anxiety surrounding GI symptoms. Youth were asked to indicate how strongly they related to statements regarding abdominal pain and related symptoms on a Likert scale (e.g., “I often fear that I won’t be able to have a normal bowel movement,” and “No matter what I eat, I will probably feel uncomfortable.”) ranging from 0 = strongly disagree to 5 = strongly agree. Higher scores indicate greater GI-specific anxiety. The VSI has yielded high levels of internal consistency in the past (e.g., [19,20]), as was found in the current study (α = 0.95).

Illness stigma surrounding IBS was calculated via the *Stigma Scale—Child* (SS-C [13]). The SS-C is an 8-item scale that was developed for youth with inflammatory bowel disease (IBD) and was adapted with permission for use in pediatric IBS. All questions remain the same as the original; however, any mention of “IBD” was replaced with “IBS” in the current study (e.g., “How often do you keep your IBS a secret from other kids?”). Participants rated how often (1 = “Never” to 5 = “Very Often”) they perceived their IBS as stigmatizing in different contexts. Sum scores are calculated with higher values indicating greater illness stigma. The original SS-C has continually demonstrated high levels of internal consistency (e.g., [13,21,22]) and the adapted version in the current study yielded good internal consistency (α = 0.84) at time 1.

*The Illness Intrusiveness Scale—Child* (IIS-C [23]) is a 12-item measure investigating how much an illness and its treatment disrupt activities and relationships (0 = “Does not apply to me,” to 7 = “A lot”). A total score is derived from summing items together, with higher scores indicating greater perceived illness intrusiveness. The IIS-C has demonstrated good-to-excellent internal consistency in other illness populations with intermittent disease courses (e.g., [22,23]), as it did in the current study (α = 0.90).

Illness uncertainty surrounding all aspects of an illness from diagnosis, treatment, to prognosis (e.g., “I have a lot of questions about my IBS and don’t know what the answers are,” “It’s hard to know if the treatments or medicine I am getting are helping me get better.”) was measured using the *Child Uncertainty in Illness Scale* (CUIS; Mullins & Hartman, unpublished manuscript, 1995), a 23-item questionnaire. Participants indicate how true a statement is using a Likert scale ranging from “Very True” to “Very False.” Certain items are reverse scored before summing all items to obtain a total score. Higher scores on the CUIS indicate greater illness uncertainty. The CUIS has yielded good internal consistency in other pediatric health conditions (e.g., [24]), as was the case in the current study (α = 0.89).

Pain beliefs and coping styles were measured using the *Pain Beliefs Questionnaire—Short Form* (PBQ-SF [25]), an 18-item questionnaire that asks participants how true statements may be about their stomach pain on a 5-point scale (0 = “Not at all true” to 4 = “Very true”). The PBQ-SF yields scores for three domains, problem-focused coping efficacy (PBQ-PFCE), emotion-focused coping efficacy (PBQ-EFCE), and pain threat (PBQ-PT). Items pertaining to each domain are averaged together to obtain a subscale score, with higher scores indicating greater beliefs in the ability to cope with pain or that pain is perceived as a greater personal threat. The PBQ-SF has demonstrated good internal consistency in the past (e.g., [25]) and good and acceptable internal consistency in the current study (PBQ-PFCE, α = 0.88; PBQ-EFCE, α = 0.80; PBQ-PT, α = 0.75).

***Belonginess and Mood***

The 9-item *Thwarted Belongingness Subscale* on the *Interpersonal Needs Questionnaire* (INQ-TB [26]) was utilized to assess youth respondents’ perception of social isolation or connectedness to others (e.g., “These days, I feel disconnected from other people”). Each item presents a statement and asks how true it is for the individual on a 7-point Likert scale (1 = “Not at all true for me,” to 7 = “Very true for me”). Higher sum scores indicate a greater perception of thwarted belongingness. The INQ-TB has demonstrated good internal consistency in previous studies (e.g., [13,26]) and was found to have excellent internal consistency in the current study (α = 0.91).

Depressive symptoms were captured on the *Children Depressive Inventory-2 Short Form* (CDI2:SF [27]). The 12-item measure asks youth to indicate which statement (in a group of three per item) best matches how they felt in the past two weeks (e.g., “I am sad once in a while,” “I am sad many times,” or “I am sad all the time”). The higher the total score is, the greater the depressive symptoms. The CDI2:SF has yielded high internal consistency in previous work (e.g., [28]). The CDI2:SF demonstrated good internal consistency in the current study as well (α = 0.85).

*The Penn State Worry Questionnaire—Child* (PSWQ-C [29]) is a 14-item questionnaire that has statements about anxiety (e.g., “When I finish one thing, I start to worry about everything else.”) and asks respondents to indicate how true a sentence is using a 4-point Likert scale (“Never true” to “Always true”). Higher sum scores indicate greater anxiety. The PSWQ-C continually demonstrates high internal consistency (e.g., [30]) and the current study was no exception (α = 0.95).

**Data Analysis Plan**

Guided by Wright et al.’s 2009 adaptation [15] of Mishel’s (1990 [16]) Antecedents and Outcomes of the Uncertainty in Illness model, antecedents comprised biological (i.e., abdominal pain, visceral sensitivity), psychological (cognitive appraisals, i.e., illness stigma, illness intrusiveness), and social factors (i.e., thwarted belonginess). Illness uncertainty served as the mediator, and outcome variables were comprised of psychological distress (i.e., depressive and anxiety symptoms), danger/opportunity (i.e., perceived pain threat), coping (problem-focused coping efficacy, emotion-focused coping efficacy), and pain interference (i.e., functional disability).

Descriptive statistics were conducted with SPSS version 28. Mediation models with baseline (T1) predictors (i.e., abdominal pain, illness intrusiveness, visceral sensitivity, illness stigma, and thwarted belongingness), 3-month (T2) mediators (i.e., illness uncertainty), and 6-month (T3) outcome variables (i.e., depressive symptoms, anxiety symptoms, problem-focused coping, emotion-focused coping, pain threat, and functional disability) were constructed with Mplus version 9 using Bayesian estimation to maintain power with a limited sample size. Multiple Imputation was used by default to handle the 4% missing data. Markov chain Monte Carlo with 50,000 iterations was used to sample the posterior distribution. Bayesian model adequacy and convergence were evaluated using multiple diagnostic indices. Overall model fit was assessed using the posterior predictive *p*-value (PPP). Convergence was evaluated using the potential scale reduction (PSR) statistic, effective sample sizes, and visual inspection of trace plots. PSR values below 1.05, effective sample sizes greater than 200, absence of Mplus convergence warnings, and stable trace plots with good chain mixing were considered indicative of adequate convergence (e.g., [31]).

Individual mediation models were developed for each outcome variable in an attempt to account for the conservative sample size and reduce the likelihood of model over-fit. Given the past literature and the sample demographics, gender, age, and IBS type (i.e., diarrhea, constipation, or mixed) were included in the models as covariates [32]. To account for the limited sample size, effect sizes were examined using STDYX standardization estimates [33] for indirect effects. The standardized estimates (β) were interpreted as 0.01 = small, 0.09 = medium, and 0.25 = large effect sizes [34]. Given the exploratory pilot nature of the study and limited sample size, indirect effects were interpreted with consideration for effect magnitude, confidence intervals, and hypothesized direction rather than statistical significance alone.

## 3. Results

**Descriptives**

Participants (N = 50) were on average 15 years old (M = 14.50 years old, SD = 2.38 years), and a majority identified as female (80%). Most participants were white (94%) and non-Hispanic (98%; see Table 1 for more details). Regarding their IBS, 74% had IBS—constipation type, followed by 16% mixed type, and 10% diarrhea type. At baseline assessment (T1), nearly half of the participants had active symptoms, whereas at the 3-month (T2) and 6-month (T3) follow-up, the majority of participants (64%) had dormant symptoms. Prescribed IBS treatment included oral prescription (82.51%), mental health counseling (47.94%), low-FODMAP diet (37.82%), physical therapy (12.77%), and IB-STIM (11.59%). See Table 2 for correlations between variables.

**Mediation Models**

*Model Performance*

Bayesian estimation diagnostics indicated adequate model performance across analyses. Posterior predictive *p*-values were acceptable (PPP = 0.167), PSR values were below 1.05, effective sample sizes exceeded 200 for all estimated parameters, no convergence warnings were generated by Mplus, and visual inspection of trace plots demonstrated satisfactory chain mixing and convergence.

*Direct Effects*

Across all mediation models, there were no significant T1 predictors of T2 illness uncertainty (a path). However, T2 illness uncertainty significantly predicted T3 depressive symptoms (β = 0.35, 95% CI [0.08, 0.59]), anxiety symptoms (β = 0.45, 95% [CI 0.19, 0.66]), problem-focused coping (β = −0.38, 95% CI [−0.61, −0.11]), emotion-focused coping (β = −0.34, 95% CI [−0.58, −0.05]), and functional disability (β = 0.37, 95% CI [0.10, 0.59]) (b path).

*Indirect Effects*

There were no statistically significant indirect effects via illness uncertainty (c’ path).

*Exploratory Indirect Effects*

**Figure 1:**
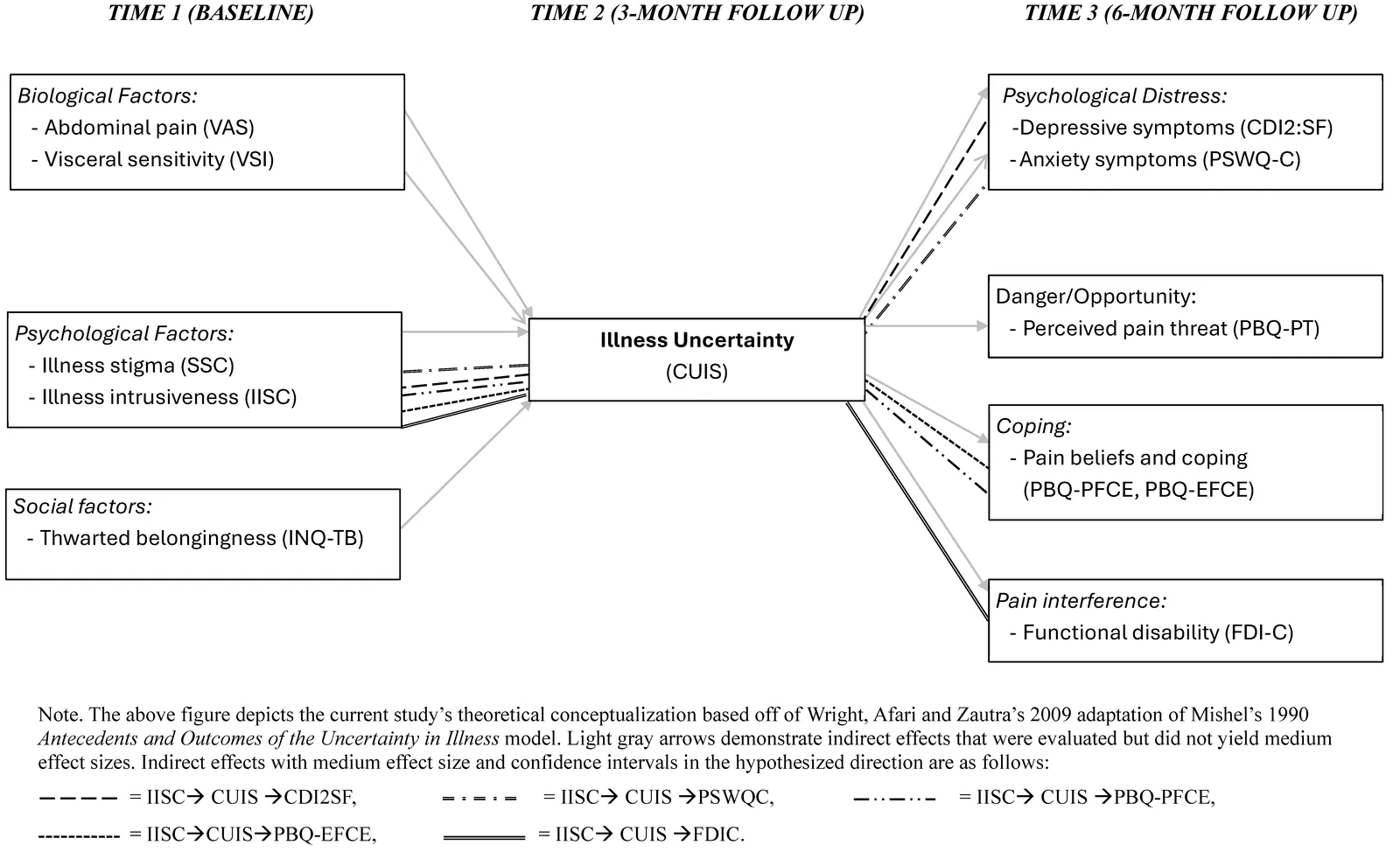
**Mediation Models** [15,16].

Although none of the indirect effects reached conventional statistical significance, illness uncertainty demonstrated indirect effects of moderate magnitude in the expected direction for the relationship between illness intrusiveness and depressive symptoms (β = 0.10, 95% CI [−0.04, 0.24], *p* = 0.16), anxiety symptoms (β = 0.13, 95% CI [−0.03, 0.29], *p* = 0.11), problem-focused coping (β = −0.11, 95% CI [−0.25, 0.03], *p* = 0.13), emotion-focused coping (β = −0.10, 95% CI [−0.23, 0.04], *p* = 0.17), and functional disability (β = 0.10, 95% CI [−0.04, 0.24], *p* = 0.14). See Figure 1 for visual depiction.

## 4. Discussion

This longitudinal pilot study examined the role of illness uncertainty in pediatric IBS. Baseline (T1) predictors of interest (e.g., abdominal pain, illness intrusiveness, visceral sensitivity, illness stigma, and thwarted belongingness) did not significantly predict illness uncertainty 3 months later (T2). However, increased levels of illness uncertainty did predict higher levels of depressive and anxiety symptoms and functional disability and less problem-focused and emotion-focused coping. Despite the indirect effects not being statistically significant, further exploration of indirect effect estimates with medium effect sizes and confidence intervals in the expected direction suggested that illness uncertainty may be a meaningful pathway linking illness intrusiveness and psychological and functional outcomes in pediatric IBS. These pathways warrant continued investigation in studies designed with sufficient power to detect effects of this magnitude, as it is plausible that they will result in statistical significance.

Indeed, previous studies in other GI populations have found significant direct and indirect effects between illness intrusiveness and/or illness uncertainty and mood (e.g., [35,36]). In the current study, illness intrusiveness (IIS-C) emerged as the most consistent independent variable in the mediation pathways, with illness uncertainty (CUIS) being the intermediary variable and depressive and anxiety symptoms serving as outcome variables (i.e., IIS-C→CUIS→ CDI2:SF; IIS-C→CUIS→PSWQ-C), yielding a medium effect size. This may indicate that the pervasive thoughts surrounding IBS’s negative impact on daily activities, and/or modifications needed to accommodate symptoms, coupled with the difficulty identifying when a flare will occur, are emotionally taxing, thus contributing to further declines in mood. Replication in adequately powered samples would help clarify whether these pathways hold at conventional significance thresholds. It is also recommended that thwarted belongingness be examined for its potential to serve as a moderator.

Beyond its association with mood, illness uncertainty also appeared to extend into functional and pain-related domains in the exploratory analyses. In those who perceive their IBS as intrusive, the inherent uncertainty surrounding it may lead to anticipatory dread regarding future occurrences of a symptom flare, which may then eventuate into increased functional disability (i.e., IIS-C→CUIS→FDI-C). Interestingly, in the current sample, youth’s reports of abdominal pain (VAS) did not predict illness uncertainty, yet the indirect relationship between the appraisal that IBS interferes with life domains (IIS-C) and perception of pain as a personal threat (PBQ-PT) with illness uncertainty (CUIS) as the mediator was observed to have a small effect size with confidence intervals in the right direction (i.e., IIS-C→CUIS→PBQ-PT, β = 0.07, 95% CI [−0.02, 0.23], *p* = 0.27). Given that the confidence intervals were trending in the appropriate direction, this pathway may merit further attention in future work despite not meeting the medium effect size threshold used to guide interpretation in the current study.

The heightened uncertainty and pain threat appraisal may also help explain the pattern of treatment adherence in this population. In our previous work, youth reported only taking their prescribed supplements and medications during a symptom flare, as opposed to preventatively (e.g., [37]), which may be due to the surrounding stigma and uncertainty of IBS, subsequently resulting in increased symptom severity. Collectively, these pilot findings demonstrate the importance of assessing cognitive appraisals even in the absence of disease symptoms, as they may serve as meaningful points of intervention to reduce symptoms or at least symptom severity. The present study expands on several findings from the adult IBS literature demonstrating the negative impact illness-related appraisals have on visceral sensitivity, psychological distress, coping, and quality of life (e.g., [10,38,39]). Together, these observations suggest that illness uncertainty may represent a critical mechanism through which IBS-related experiences and appraisals contribute to emotional and functional outcomes, thus highlighting the importance of targeting such appraisals, particularly illness uncertainty, in future interventions.

Regarding risk and resilience factors, it is plausible that both the indirect and direct effects that CUIS had on PBQ-PFCE and PBQ-EFCE with (indirect) or without IISC’s presence (direct effect) are also worth further investigation. In the current pilot study, higher illness uncertainty led to lower problem- and emotion-focused coping efficacy or confidence in one’s ability that they can reduce their pain and confidence in one’s ability that they can mentally or emotionally adapt to their pain, respectively. One means of reducing illness uncertainty is to provide patients and families with greater neurogastroenterology education surrounding IBS, including the mind–body connection, as well as helping to destigmatize this relationship and subsequently empowering youth with evidence-based coping skills that will promote their confidence in their ability to change their mood or act on their pain in the presence of IBS. In accordance with current recommendations [40], having pediatric psychologists as part of the GI treatment team would lend itself to these points of intervention. Further, having other team members engage in positive dialog with the patient and caregivers about the pediatric psychologist’s role will likely contribute to more positive connotations of collaboration, increased treatment buy-in, and decreased stigma of working with a psychologist. On a larger scale, additional and ongoing didactics surrounding neuroscience education and the importance of utilizing a biopsychosocial approach in clinical care should occur amongst medical providers and clinical support staff. This is just one example of a larger systems approach that is necessary to help continuously promote best practices of care and enable patients to be better advocates for themselves.

Several limitations should be considered when interpreting these findings. First, this was an exploratory pilot study with a modest sample size, which may limit the precision and stability of parameter estimates and increase the risk of overfitting despite efforts to minimize model complexity through a priori model specification and inclusion of only essential covariates. Consequently, these findings should be viewed as preliminary and hypothesis-generating to help inform larger future studies. Lastly, the current sample is fairly homogeneous (e.g., predominantly female, white, IBS-C). Although these characteristics are consistent with a number of previously reported pediatric IBS cohorts in the literature (e.g., [32,41,42,43]), it may impact the generalizability of the findings to broader cohorts. Future studies with larger, adequately powered, and diverse samples are needed to further evaluate the proposed biopsychosocial model of illness uncertainty in pediatric IBS through more comprehensive path analyses that can simultaneously examine the interrelationships among antecedents, illness uncertainty, and multiple health-related outcomes. One potential extension may be to incorporate baseline outcome levels as covariates in mediation models to examine whether illness uncertainty is associated with changes in symptoms and functioning over time, beyond initial levels of these outcomes.

Notwithstanding these limitations, this pilot study provides preliminary evidence supporting the potential role of illness uncertainty as a longitudinal mechanism linking biopsychosocial factors with psychological and functional outcomes in youth with IBS. These findings provide an important foundation for future research aimed at validating these pathways in larger, adequately powered studies.

## Figures and Tables

**Table 1:** **Descriptive Statistics (N = 50)**.

Variable	N (Valid %)
*IBS type*	
Constipation	37 (74)
Diarrhea	5 (10)
Mixed Constipation and Diarrhea	8 (16)
*Race*	
White	47 (94)
Black	1 (2)
Asian American/Pacific Islander	1 (2)
Biracial/Multiracial	1 (2)
*Ethnicity*	
Non-Hispanic	49 (98)
Hispanic	1 (2)
*Gender Identity*	
Female	40 (80)
Male	10 (20)

**Table 2:** **Correlations**.

	1.	2.	3.	4.	5.	6.	7.	8.	9.	10.	11.	12.	13.	14.	M (SD)
1. Age	-														14.50 (2.38)
2. Gender identity	0.04	-													--
*T1*															
3. VAS	−0.11	0.03	-												5.32 (2.19)
4. IIS-C	0.17	0.04	**0.44**	-											27.76 (17.55)
5. VSI	0.20	**0.29**	0.14	**0.42**	-										36.24 (18.06)
6. SS-C	0.19	0.06	0.17	**0.54**	**0.54**	-									19.06 (6.56)
7. INQ-TB	0.14	−0.07	0.03	**0.39**	0.26	**0.53**	-								24.88 (12.52)
*T2*															
8. CUIS	−0.01	0.06	0.24	**0.52**	**0.42**	**0.44**	**0.30**	-							66.55 (13.62)
*T3*															
9. CDI2:SF	0.16	0.01	0.20	**0.45**	0.19	**0.44**	**0.68**	**0.41**	-						6.88 (4.55)
10. PSWQ-C	0.01	0.13	0.16	**0.39**	**0.39**	**0.45**	**0.33**	**0.52**	**0.46**	-					24.42 (10.84)
11. PBQ-PFCE	−0.15	−0.16	−0.21	−0.18	**−0.33**	−0.28	−0.16	**−0.44**	**−0.43**	**−0.29**	-				10.13 (5.46)
12. PBQ-EFCE	0.22	0.06	−0.04	−0.05	−0.26	−0.07	−0.18	**−0.40**	**−0.41**	−0.25	**0.51**	-			16.25 (4.82)
13. PBQ-PT	0.25	0.19	0.13	0.16	0.26	0.18	0.28	**0.31**	**0.55**	0.26	**−0.66**	**−0.59**	-		9.54 (4.90)
14. FDI-C	0.12	0.07	**0.32**	**0.57**	0.24	**0.40**	**0.41**	**0.43**	**0.70**	0.26	**−0.47**	**−0.37**	**0.45**	-	13.44 (11.31)

Note. **Bold** = significance at least *p* < 0.05. Age mean and standard deviation are listed in years. T1 = time 1, T2 = time 2, T3 = time 3. VAS = visual analog scale pain rating, IIS-C = illness uncertainty, VSI = visceral sensitivity, SS-C = illness stigma, INQ-TB = thwarted belongingness, CUIS = illness uncertainty, CDI2:SF = depressive symptoms, PSWQ-C = anxiety symptoms, PBQ-PFCE = problem-focused coping efficacy, PBQ-EFCE = emotion-focused coping efficacy, PBQ-PT = pain threat, and FDI-C = functional disability.

## Data Availability

Due to multiple institutions’ involvement, data management policies, and because this is an understudied pediatric population at risk for identification, the dataset is not available for public use.

## Abbreviations

The following abbreviations are used in this manuscript:

AMA	American Medical Association
APA	American Psychological Association
CDI2:SF	Child Depressive Inventory 2nd Edition—Short Form
CUIS	Child Uncertainty in Illness Scale
FDI-C	Functional Disability Index—Child
GI	Gastroenterology
IBD	Inflammatory Bowel Disease
IBS	Irritable Bowel Disease
IIS-C	Illness Intrusiveness Scale—Child
INQ-TB	Interpersonal Needs Questionnaire—Thwarted Belongingness Subscale
PBQ-EFCE	Pain Beliefs Questionnaire—Emotion-Focused Coping Efficacy
PBQ-PFCE	Pain Beliefs Questionnaire—Problem-Focused Coping Efficacy
PBQ-PT	Pain Beliefs Questionnaire—Pain Threat
PSWQ-C	Penn State Worry Questionnaire—Child
SS-C	Stigma Scale—Child
T1	Baseline
T2	3-Month Follow-Up
T3	6-Month Follow-Up
VAS	Visual Analog Scale
VSI	Visceral Sensitivity Index
